# The Light Node Communication Framework: A New Way to Communicate Inside Smart Homes

**DOI:** 10.3390/s17102397

**Published:** 2017-10-20

**Authors:** Valère Plantevin, Abdenour Bouzouane, Sebastien Gaboury

**Affiliations:** Département d’Informatique et de Mathématiques, Université du Québec à Chicoutimi, Saguenay, QC G7H 2B1, Canada; abdenour_bouzouane@uqac.ca (A.B.); sebastien_gaboury@uqac.ca (S.G.)

**Keywords:** messaging protocol, IoT, smarthome, distributed computing

## Abstract

The Internet of things has profoundly changed the way we imagine information science and architecture, and smart homes are an important part of this domain. Created a decade ago, the few existing prototypes use technologies of the day, forcing designers to create centralized and costly architectures that raise some issues concerning reliability, scalability, and ease of access which cannot be tolerated in the context of assistance. In this paper, we briefly introduce a new kind of architecture where the focus is placed on distribution. More specifically, we respond to the first issue we encountered by proposing a lightweight and portable messaging protocol. After running several tests, we observed a maximized bandwidth, whereby no packets were lost and good encryption was obtained. These results tend to prove that our innovation may be employed in a real context of distribution with small entities.

## 1. Introduction

The evolution of our society towards the all-digital Internet of things (IoT) has profoundly remodeled our relationship with the science of information. In accordance, the smart home has become the subject of numerous studies [1,2,3] and joins a recent current of thought stemming from ambient intelligence (Amb. I). The latter concept refers to a tendency towards miniaturizing a set of electronic devices (sensors and effectors) in order to integrate them into any object used on an everyday basis (lamps, refrigerators, etc.) in a transparent way for individuals. The aim behind this idea is to supply timely assistance to the occupants according to the gathered information and to the history of the accumulated data.

The vast majority of work with respect to the domain of smart homes focuses on an activity recognition problem in order to potentially assist inhabitants with dementia often caused by advanced age [4,5,6]. Nevertheless, none of the studies seem to propose a standard architecture providing both high reliability and scalability capabilities at a relatively low cost. High-reliability is a mandatory feature of such architecture since assistance is vital for inhabitants with potential dementia. Moreover, as the disease can remain for decades, any work on architecture must take a scalability parameter into account since many changes in sensors or improvements can be made over the evolution of an illness. Finally, the low-cost aspect has to be taken into account as the vast majority of the aging population will be located in poor or developing countries by 2050 [7]. As far as we know, this paper is the first focusing on those three points in particular.

Here, we briefly introduce a new kind of smart home architecture providing both reliability and scalability based on low-cost smart sensors. To achieve this objective, the first issue we ran into was the difference between all the possible entities in the environment. In fact, our solution has to integrate different operating systems (e.g., Linux or FreeRTOS) running on different types of hardware (e.g., computers or micro-controllers) and using different communication technologies (e.g., Wi-Fi, ZigBee or 6LowPan). To respond to these dissimilarities, we have to use a highly portable communication protocol using brokerless architecture to provide the greatest reliability. This point will be the main concern of this paper. Even if many protocols exist, like Message Queue Telemetry Transport (MQTT), RabbitMQ, or ZeroMQ [8,9,10], none of them fully respond to our requirements since the first two protocols require brokers to work and the last one is based on Portable Operating System Interface (POSIX) sockets and cannot be embedded in some light systems. Consequently, the contribution we make in this paper is a new way to communicate that can be embedded in each system as soon as it implements an Internet Protocol (IP) stack. Our solution provides a discovery mechanism, security via Advanced Encryption Standard (AES) encryption, and two different channels in order to address the difference between configuration messages and data messages. This paper is divided into four sections. The first section presents the state- of-the-art with respect to existing smart homes and their architectures. The second part will cover the technological breakthroughs that embedded computing has experienced since the creation of the first smart homes. Then, the proposed solution will be explained and some tests on the messaging protocol will be presented in the third section. Finally, conclusions and some future works are found at the end of this paper.

## 2. Existing Architectures

Smart habitations have been implemented in laboratories since the creation of ambient intelligence [1,3,11,12]. Each of these projects uses the technology of its day to create a testing environment in which the data accessibility was the main challenge. Here, we depict three of these projects, starting with the *Labotatoire d’Intelligence Ambiante pour la Reconnaissance d’Activités* (LIARA) and *labotatoire de Domotique et informatique mobile à l’Université de Sherbrooke* (DOMUS), which share the same architecture [12]. We continue this review with the Gator Tech house [3] and the Center for Advanced Studies in Adaptive Systems (CASAS) [1]. Finally, we end this part by describing a software-defined smart home [13,14], a recently released type of architecture based on software-defined networks.

### 2.1. LIARA and DOMUS

**Figure 1:**
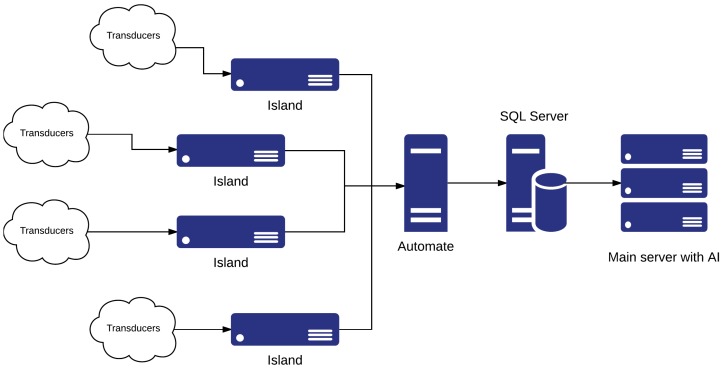
The *Laboratoire d’Intelligence Ambiante pour la Reconnaissance d’Activités* (LIARA) and *Laboratoire de domotique et Informatique Mobile à l’Université de Sherbrooke* (DOMUS) architecture. AI: artificial intelligence.

LIARA and DOMUS laboratories aim to study how smart homes can assist people with cognitive deficiencies. They use very similar architecture, represented in Figure 1, to test their algorithms and solutions [15]. Inherited from the industry, they use some islands, made of industrial-grade hardware, to agglomerate transducers. Then, an automate is in charge of getting back, from the islands, the values of the sensors or changing the values of the effectors. To end this process, the automate will update a relational database hosted on an *SQL Server* in order to provide a simple interface for other systems like, in this case, artificial intelligence. These two smart homes present some interesting features we have to discuss. First, the use of industrial-grade hardware means that all the components have been tested for continuous use in a far more complex environment than just a house (e.g., a production line in a factory). Therefore, excellent reliability is demonstrated even if the smart home has to operate at all times. The second main advantage of these environments comes from the highly centralized architecture itself. Indeed, all the values coming from sensors and all the actuator controls end up in the same database. As a result, interaction is facilitated with the home since this kind of storage offers an easy way to retrieve sensor values or interact with actuators.

Nevertheless, the industrial material suffers from two main drawbacks. The first one is the introduction of black boxes in a research environment. As a matter of fact, the communications between all these pieces of hardware often rely on proprietary libraries that can impact future evolution. The second main disadvantage is the price of such architecture. Based on the hardware presented by Bouchard et al. [15] and the price, we were able to compute the total price of the island–automate–main server chain. With costs of USD 2000 for each island [16], USD 1500 for the automate [16] and another USD 4000 for the server [17], the architecture cost reaches 13,500 dollars without any transducers or backbone structure (e.g., networking, cooling for the server, maintenance). Finally, the highly centralized architecture presented here creates many single points of failure (SPoFs) like the automate, the islands, and the main server hosting both the artificial intelligence (AI) and the SQL server. Hence, if one of these SPoFs fails, at least a quarter of the environment and its assistance will fail too. Moreover, these points represent some serious bottlenecks in the architecture, preventing real scalability.

### 2.2. Gator Tech

**Figure 2:**
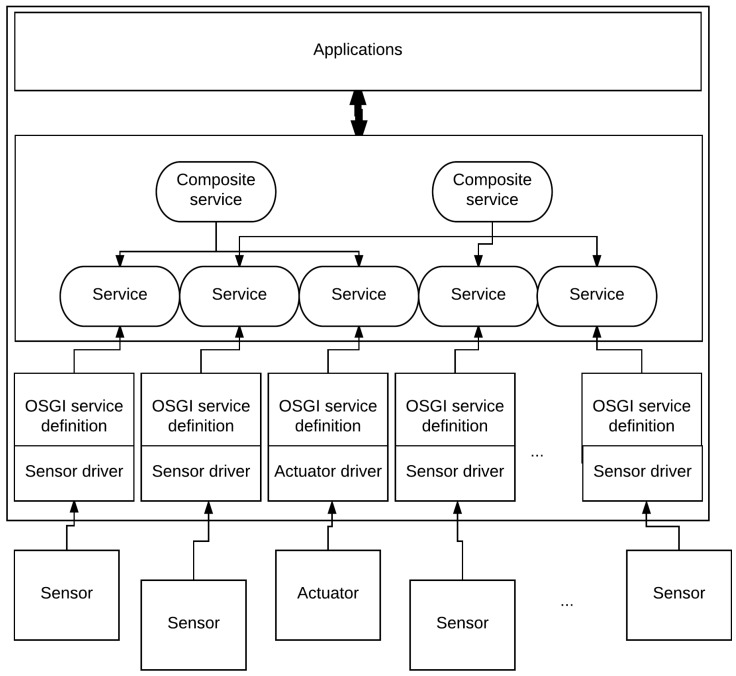
The Gator Tech architecture. OSGI: Open Service Gateway initiative.

Gator Tech [3] is a project based in Florida. Its main goal is to prove the feasibility of a low-cost smart home where the integration of new transducers will be easy. To this end, the authors present an Open Service Gateway initiative (OSGI)-based architecture [18] that is summed up in Figure 2. Each transducer has a simple electrically erasable programmable read-only memory (EEPROM) containing the driver to communicate with it. Once powered, the transducer registers itself by sending its driver for OSGI service definition. This will act as an abstraction layer to create basic services that allow the consumption of highly abstracted data (e.g., “sunny” instead of 10,000 lumen for a light sensor) or the combination of basic services for a composite service (e.g., the creation of a voice recognition service using all the different microphone services). All this architecture allows developers to create applications without any knowledge of the underlying communication and with only highly abstracted data, which simplifies development.

This environment has some very good advantages. First of all, the automatic transducer registration helps the scalability of such an environment (e.g., the addition of new sensors or replacement of some of them). Secondly, the high abstraction of the data generated by this system greatly helps the application development. For example, it is straightforward to enable air conditioning when the temperature is “hot”. However, it is more complicated when the decision is only based on the micro-controller value since this depends on the hardware (e.g., the micro-controller itself, the temperature sensor, or even the analog to digital chips). Finally, the price of such infrastructure is as low as possible as each transducer is designed to be the most affordable by using Atmega128 as the main processor unit, which is a low-cost platform [19]. Moreover, because every transducer is wireless, there is no need for islands or automates, as in the LIARA and DOMUS homes. Despite all these great advantages, the use of OSGI on a unique server creates an SPoF, which is a big problem in high-reliability architecture.

### 2.3. CASAS

**Figure 3:**
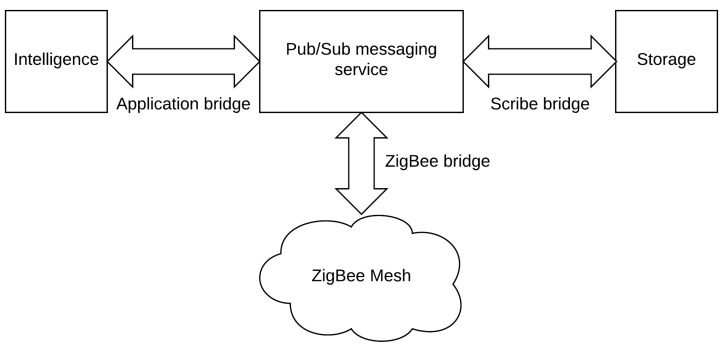
The CASAS architecture. pub/sub: publish/subscribe.

CASAS [1], also known as the "smart home in a box", is an infrastructure where the emphasis is put on the price and ease of installation. Depicted in Figure 3, the architecture is divided into four main elements. The first one is the ZigBee mesh which represents all the transducers communicating between them in a network where each node relays the information to its neighbors using the ZigBee protocol. This mesh sends events using a publish/subscribe (pub/sub) messaging service through a ZigBee bridge in charge of converting events to higher-level eXtensible Messaging and Presence Protocol (XMPP) messages. The messaging service allows other applications to easily integrate the infrastructure and use the transducers. By default, there are two services: storage and intelligence. The first archives all the events occur in the environment by using the Scribe bridge. As for the intelligence, it is in charge of energy monitoring and the discovery and recognition of any activity that can happen in the house.

CASAS has two main benefits: price and the ease of installation. For the former, the authors present a detailed summary of the cost. They state that their solution costs only *USD 2765* , which is a great achievement. Regarding the ease of installation, they demonstrate this by conducting a test on people aged from 21 to 62. Only an hour is required to set up the whole environment. In spite of these qualities, CASAS, as with other architecture, suffers from the existence of many SPoFs like the ZigBee bridge or the Application bridge which can halt the assistance or the pub/sub-messaging service, which is a sensitive component.

### 2.4. Software-Defined Smart Home

The works previously described are the old founders of smart home architecture. However, some more recent papers exist in this particular domain [13,14]. One study introduces the idea of the software-defined smart home or SDSH for short [13]. This concept is a new way to integrate heterogeneous smart appliances (e.g., smart lights, smart flowerpots, etc.) in one homogeneous platform, creating a smart home from the chaos of the different hardwares and communication protocols implemented by the companies who create these devices. To achieve such a goal, the authors propose a three-layer architecture derived from software-defined networks [20]. First, the smart devices layer includes all the different kinds of smart hardware in a home (also known as smart appliances). Next, the controller layer is a centralized management service locally implemented or deployed in the cloud. Its main goal is to hide the implementation details of the hardware layer, retrieve and analyze the user demands, and manage the whole smart home. Moreover, it is in charge of encapsulating information extracted from the smart home and providing them to the last layer: the external service layer. This layer uses the smart home resources to provide some smart services like home security or medical attention.

SDSH offers some great features. First of all, as with Gator Tech, this architecture offers a strong separation between raw sensors and final services via its controller layer. Next, it uses OpenFlow [21] as its main protocol which is a well-known protocol widely implemented in software-defined networks. Finally, it uses smart appliances already on the market and standardizes the access to their data. Unfortunately, the centralized controller depicted as one of the main advantages of this work is also a great disadvantage because, if it allows for configuration of the whole smart home in the same place, it represents a severe single point of failure. To address this problem, the authors introduced visualization techniques but these require either an Internet cloud connection (which cannot be tolerated in some applications) or a server strong enough to deal with many systems started on the same hardware which can be very expensive for a single house.

### 2.5. Conclusions on Existing Architectures

All these existing architectures have some common points. First, the majority of their components are transducers. Moreover, according to the Gator Tech and CASAS cases, it seems that embedding some intelligence and communication abilities in them helps to reduce costs and ease installation and scalability. Finally, we have to point out the common problem in all these architectures: centralization. This weakness creates single points of failure which can lead to a complete halt in assistance. In corporate computing and Web domains, this particular issue was solved ten years ago by using redundancy, clusters and distributed computing [22,23,24,25].

The ideal architecture appears to be composed of many smart transducers easing the scalability of such architecture. This specific attribute brings our environment closer to another computer science domain, which is the Internet of things (IoT). In IoT, already used in smart homes [26,27], a multitude of smart objects communicate in a uniform manner, generating a huge amount of data often associated with “big data” [28]. In this situation, it is not conceivable to handle the information in a centralized way any more, even if we use server clusters. It is more appropriate to use decentralized methods relocating the intelligence as close as possible to the units comprising this huge data pool [28,29].

## 3. Technological Breakthroughs

The transducers (i.e., sensors and actuators) form the essential basis of all smart home architecture. CASAS and Gator Tech case studies proved that embed intelligence and communication in these entities allow for a reduction in costs while improving the ease of implementation and the scalability. In this part, we are going to study the concept of a smart transducer such as that designed by the standards. Then, we will review part of the hardware evolution that has taken place since the creation of the first smart homes.

A smart transducer is clearly defined in the Institute of Electrical and Electronics Engineers (IEEE) 1451.2 standard [30]. To sum up, it is an entity providing more features than is mandatory to generate a good representation of the controlled quantity. Some of these attributes can include sensor identification, a process to simplify the installation or the maintenance, network interfaces, or coordination and synchronization with other entities [31]. In order to guide the community, Lewis [31] proposed three objectives for these transducers. The first one is to move the intelligence closest to the sensing point. The second objective is to make the installation easier, and the maintenance of massive distributed sensor networks less expensive. The last objective is to facilitate the interface of many different sensors. Now that the concept of a smart transducer is well defined, we can work with the different technological breakthroughs that have occurred over the last ten years and that allow us to finally design and build inexpensive smart sensors for smart environments.

Many prototypes of smart environment have emerged over the last decade (e.g., Gator Tech in 2005 or LIARA/DOMUS in 2009 [3,12]). They have been built on top of existing technologies, which for the most part are anterior to the great innovations made recently. One of these is the apparition and in particular the democratization of the system-on-chip (SoC) which are full systems integrated on a single substrate providing all the elements needed to run an application (e.g., processor, memory, radio). SoCs are the cutting edge of modern electronics and can be found everywhere, from smart sensors to nano-computers, driving price and power consumption reduction in all modern devices [32].

To illustrate the growth in power and integration, we propose a quick comparison between two micro-controller platforms and some nano-computers. The first two are the Arduino USB, easily accessible at the time of the creation of the first smart homes, and the recently released ESP 32 from the Espressif company. With respect to nano-computers, we chose the evolution of the Raspberry Pi computer since its creation. The attributes we compare are the released year, the processor frequency, the memory available, the connectivity, the relative size, and the price. Table 1 and Table 2 represent the different values for the attributes retained for the micro-controllers and the Raspberry Pi, respectively. The first feature that can be noted from these tables is the increase in both processor frequency and memory, with 16 MHz and 1 kB of Random Access Memory (RAM) for the Arduino USB, and a dual core of 240 MHz with 512 kB of RAM for ESP 32. The phenomenon is the same for the different forms of the Raspberry Pi nano-computer, with 700 MHz and 512 MB of RAM for Pi 1, and 1 GHz and the same amount of memory but with half the size for Pi Zero W, and four cores at 1.2 GHz with 1 GB of RAM for Pi 3, but with the same form factor.

Moreover, it is fairly obvious that the embedded connectivity became a must in this period with the integration of Wi-Fi and both Bluetooth and Bluetooth Low Energy (BLE) in ESP 32 and Wi-Fi/BLE for Pi Zero W and Pi 3. Finally, it must be noted that the prices of these platforms stay the same or decrease drastically even if the platforms increase in power and connectivity.

Technological evolution has been impressive since the beginning of the 2000s. The democratization of the SoC permits an increase of power for such pieces of hardware while reducing costs and power consumption. In parallel, SoCs integrate much more advanced features like Wi-Fi and Bluetooth communication. Subsequently, it seems now possible to create powerful applications on embedded hardware and one of these applications is the creation of more intelligent transducers as depicted in the IEEE 1451 standard.

## 4. Proposed Solution

We saw that existing smart homes had some weaknesses in both reliability and scalability. However, these kinds of weak points are not acceptable in the assistance domain. Here, we propose a new kind of architecture using the latest technological advances to provide a reliable and scalable distributed environment to safely run the assistance.

The main concept behind our solution is that the only non-removable elements of a smart environment are the transducers themselves. If we think about it, they represent a vast number of distributed entities. With the latest hardware innovations it is feasible to equip each of them with an intelligent entity with both communication and processing capabilities, creating a huge network with highly distributed computation potential and no single point of failure. In this vision, the generic smart entities have to respond to the three main objectives firstly formulated by Lewis [31]. This means that they must allow for movement of the artificial intelligence to the closest sensing point, provide methods for easy installation, configure and maintain the smart network, and finally ease the interface between many different sensors. The first issue that such architecture has to deal with is the difference between all the intelligent entities we can use. Indeed, if we want our solution to be the most generic possible we have to be able to cope with the most different hardware and operating systems. Thus, we want to make the integration of sensors based on different operating systems (e.g., Linux or FreeRTOS) implemented on different hardware feasible, but we should also be able to establish an interface between different communication protocols (e.g., ZigBee, Wi-Fi or BLE). In order to respond to this problem, we must find a new way of communication between all these entities.

### 4.1. Communication Protocol

There are many ways to communicate using messages in the literature or industry. As far as we know the most popular methods are MQTT, RabbitMQ and ZeroMQ [8,9,33]. MQTT is mainly used in the Internet of things applications due to its high portability and its reduced footprint in terms of memory and power. It is a publish/subscribe protocol where clients connect to a centralized instance named a broker. It supports different type of quality of service which affects the reliability of communication (the message is delivered at most once, at least once, or exactly once). Finally, it can support a “last will and testament” (LWT) which allows for the sending of a specific message on a specific subject when the entity disconnects in an abnormal way from the network. RabbitMQ, on the other hand, is a leading messaging protocol mainly used in distributed architectures. It implements the Advanced Message Queuing Protocol (AMQP) and consequently has a broker architecture which provides ease of development in favor of scalability and speed since the broker adds latency and treatment, and the messages exchanged are fairly large. Finally, ZeroMQ is a messaging system which allows developers to create the architecture themselves, including brokerless ones. The main problem with this approach is portability, since ZeroMQ relies on Portable Operating System Interface (POSIX). sockets which are only supported in Unix and Windows operating systems. To conclude, none of the leading messaging protocols fit in our application since we want one that is without a centralized unit (like a broker) and that is very portable in order to deal with the most part of the possible entities in a smart home which can be composed of embedded systems running on top of different real-time operating systems (RTOS) like FreeRTOS or RiotOS.

The contribution we make in this paper is a new communication protocol with two main characteristics. First, it can be embedded on any device from computers to micro-controllers as long as they implement an IP stack (over Wi-Fi, 6LowPan, or ZigBee IP). Second, our protocol does not have the need for any main server (also known as a broker). This last point was an issue in the most popular solutions (e.g., MQTT). To build our solution, we made two basic assumptions. The first one is that all our messages will stay in the smart home network. The second is that User Datagram Protocol (UDP) is the minimum requirement for any device that wants to communicate over a network as it is the basis of many network configuration protocols (e.g., Dynamic Host Configuration Protocol (DHCP) or Domain Name System (DNS)).

One of the first issues we ran into is the fundamental difference between configuration and data streams. The first one has to be based on a reliable delivery system allowing point-to-point communication without the urge of the highest data speed. The second one must be able to stream a huge quantity of information in a minimum amount of time without the highest degree of reliability for many different listeners.

In order to respond to this problem, we propose the use of two different channels, like in the File Transfer Protocol (FTP) [34]. We will now explain how these two channels work in order to offer all the features we want.

#### 4.1.1. Configuration Channel

As mentioned previously, the configuration of a smart entity has to be distributed using reliable communication. In order to achieve this objective, we propose the use of the Constrained Application Protocol (CoAP), a well-known IoT protocol, already implemented in many platforms [35]. It allows us to use Hypertext Transfer Protocol (HTTP)-like requests to obtain or change values represented by URI in a fail-safe manner based on an acknowledgment system for important messages (i.e., messages with high reliability). We propose use of this URI representation for the entity configuration. In order to facilitate the understanding of such a concept, we present a simple example in Table 3. In this table, each line represents a possible configuration variable with its CoAP Unified Resource Identifier (URI), the methods allowed in order to get or set the information, and the type of data that is asked for by the entity. The first line is pretty obvious and represents the update frequency used by a potential sensor. It is a simple integer, represented by the URI/rate and can be obtained or modified by using respectively GET or POST requests define in the Coap protocol. The usage of an HTTP-like protocol allows us to define read-only values like the version which is a string that is only reachable via a GET request of the URI version. Moreover, we can exchange much more complex data types like JavaScript Object Notation (JSON) to clearly define hardware configurations and interactions. Another interesting feature of CoAP that we use in our configuration sample is the block-wise extension of the protocol that permits the transfer of large binary file like updates for the embedded software. Finally, the last feature of CoAP we use in this configuration channel is the ability to encrypt the communication by the usage of Datagram Transport Layer Security (DTLS). To demonstrate the utility of such a feature, we propose changing the symmetric encryption keys of the device. This kind of operation is critical since it has to be highly secure in order to guarantee that nobody can intercept these keys to listen and speak over a secure network. With our method, we simply exchange keys by using CoAP protected by Transport Layer Security (TLS) which guarantees both confidentiality and authentication. Now that the configuration channel operation is explained, we can think about how to exchange information through the data channel.

#### 4.1.2. Data Channel

In addition to a reliable communication channel, to ensure good configuration of any device, we have to provide a method to exchange data messages between the smart entities in our architecture. We want to be able to transfer huge amount of data in a minimal amount of time to discover smart sensors connected to the network and to provide the ability to encrypt sensitive information. To attain these aims, we propose a simple publish/subscribe messaging protocol based on the User Datagram Protocol (UDP) multicast chosen for its ability to transfer to one or many listeners at once in addition to its low latency. To sum up the protocol work-flow, users join the multicast group, and then they register to any topic, represented by a simple character string, and will receive any messages labeled by this topic. They may also ask for any sensors connected to the network group by sending a discovery packet with a request in it (e.g., sensors with service “temperature”) and those sensors will answer with their IP address and the different topics they expose. We are now going to explain the three different modes of our messaging protocol which are data, discovery and encrypted data.

#### 4.1.3. Data Message

**Figure 4:**
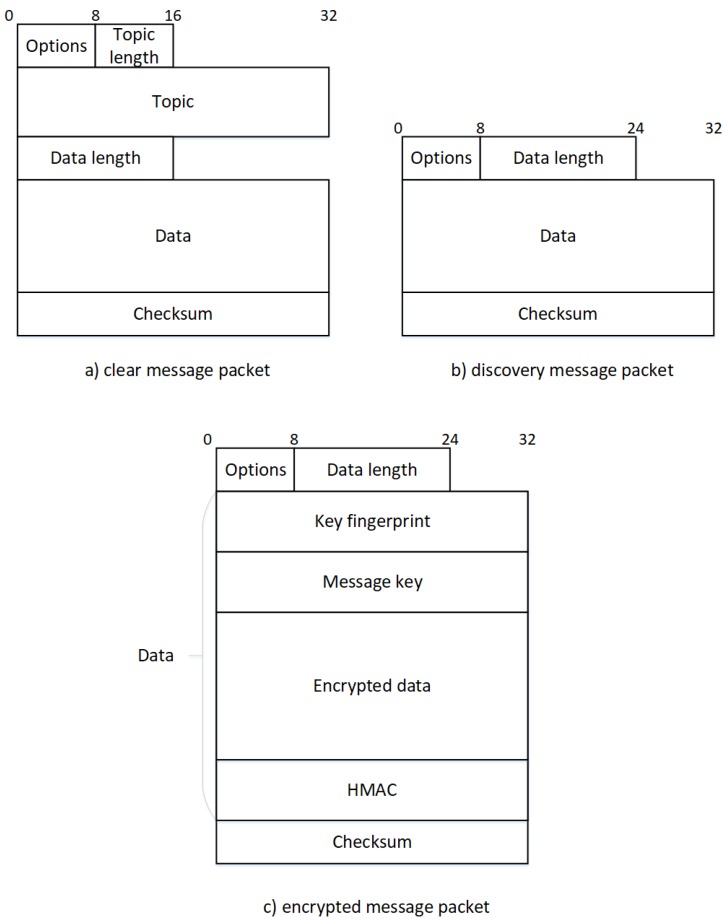
Representation of the three packets present in our protocol. Where (**a**) represents a message going through the network without encryption, (**b**) represents a standard network discovery packet and (**c**) a message going through the network using a secure algorithm to encrypt the main data. HMAC: hash message authentication code.

Figure 4a represents a single and unencrypted data message in our solution. Every packet begins with an options byte, divided into two parts. The first three most significant bits (MSBs) comprise the version number of this packet and is all set to 0 for the version we present here. The other bits are reserved and unused, except for the two least significant bits (LSBs) which represent flags used in the discovery and encrypted mode and have to be set to 0. These options are followed by the topic length, which can range from 1 to 255 characters encoded on a single byte. Any message without a topic has to be considered as an error and be forgotten. Next comes the topic with a variable length defined previously followed by the data length encoded on two bytes (the protocol tolerates empty data packages) and the data associated. Finally, a checksum is computed with the whole packet by using the cyclic redundancy check (CRC)-32 algorithm already used in the Ethernet frame which is a fast and lightweight hash algorithm. It has to be noted that the maximum size of one packet is limited by the theoretical limit of UDP which is 65,535 bytes. When a client receives a packet, the first operation to be performed is the checksum validation. If this fails, the packet has to be dropped as the protocol does not have any mechanisms to send the packet again. Otherwise, the packet can be split by using the different sizes and the data and topic can be easily read.

#### 4.1.4. Discovery Message

A really interesting feature we have to provide is a mechanism for discovering entities in the network. To achieve this, each of them can register custom key-value pairs in addition to any readable (i.e., allowing the GET method) configuration values that will be exposed to any discovery request. Figure 4b represents a single discovery packet. As the data packet, the options come first. The only difference is the LSB of this particular byte which is set to 1, representing a discovery packet. Next comes the length of the data containing the request, a simple key-value data structure represented in JSON format. For security reasons, data has to be set in order to limit the number of answers. Consequently, any discovery packets with zero-length data have to be ignored and considered as an error. When the frame is received, receivers will have to decide if they have to answer or not. To achieve this, they check every key in the request. If they do not have one of them they have to drop the packet they must not answer. Regarding the associated values, two cases are possible: either the key contains a single value or an array of values. Consequently, four situations can occur depending on the combination of two different data types for the receiver and the sender. Examples of each case are presented in Table 4. The first case shown here is when both requested and exposed data types are single values. In this case, the two values have to be exactly the same as those presented in the first two lines of the table. Next comes the case where one data type is an array and the other is a single value. Here, the single value has to be in the array (like in lines 3 to 6 in the Table 4). Finally, when both have arrays as values, at least one value in the requested array has to be present in the exposed one.

#### 4.1.5. Encrypted Data Message

As we deal with sensitive information inside a smart environment, our protocol has to provide an easy way to secure the communications. We adapt a well-known secure chat found in the Telegram [36] application to achieve this goal.

**Figure 5:**
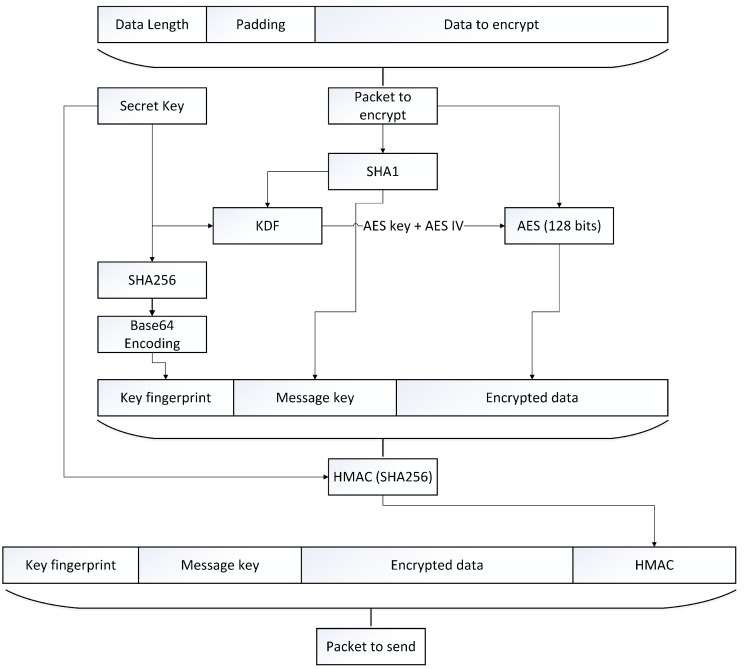
The encryption process. AES: Advanced Encryption Standard; KDF: key derivation number; SHA: Secure Hash Algorithm.

The encryption process, depicted in Figure 5, starts by adding random padding to the message. Indeed, the Advanced Encryption Standard (AES), the encryption algorithm we use, is a block cipher so our data must be a multiple of the block size $B_{s}$. The number of random bytes $P_{s}$ we have to add is defined by Equation (1). We always add $B_{s}$ bytes in order to have some randomness in the original message. Then, we add enough bytes to form a multiple of $B_{s}$ (16 in the case of AES). In Equation (1) we use DataLength+2 because the last step in the packet preparation is the prepending of the data size a two-byte-long number.

(1) $$P_{s}=B_{s}+\left(B_{s}-\left(DataLength+2\right)\%B_{s}\right)$$

When the packet is ready to be encrypted, we create the message key used to generate the AES key and initialization vector (IV) by computing the Secure Hash Algorithms (SHA)1 of the packet to encrypt. This hash is given to a key derivation function (KDF) with the secret key preconfigured by using the configuration channel. The KDF presented in Algorithm 1 is a sequence of different SHA1 hash and will result in two values, the 128-bit AES Key and AES IV used to encrypt the packet with AES. Next, in order to identify the secret key used to encrypt, we compute a unique fingerprint of it by using the Base64 representation of the key SHA256. Finally, the hash message authentication code (HMAC) is computed by using the secret key with the SHA1 hashing algorithm.

**Figure 6:**
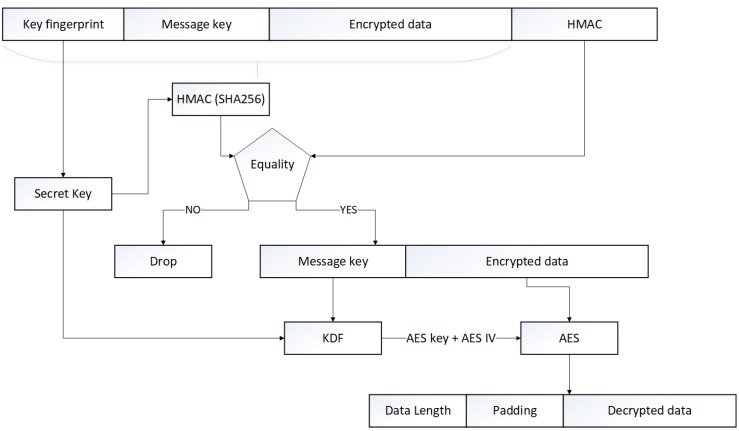
The decryption process.

**Algorithm 1:** The key derivation function**Data**: secret_key[16], msg_key[20]
**Result**: aes_key[16], aes_iv[16]
sha1_a = sha1(msg_key + secret_key[0...3]);
sha1_b = sha1(secret_key[4...5] + msg_key + secret_key[6...7]);
sha1_c = sha1(secret_key[8...11] + msg_key);
sha1_d = sha1(msg_key + secret_key[12...15]);
aes_key = sha1_a[0...3] + sha1_b[0...7] + sha1_c[4...7];
aes_iv = sha1_a[12...15] + sha1_b[12...19] + sha1_d[0...3];

In order to decrypt an encrypted packet (identified by a 1 at position 1 in the option byte of the header) the receiver has to perform some operations, as summed up in Figure 6. The first task to accomplish is the verification of the HMAC to ensure the authentication and the integrity of the packet. To accomplish this, we first have to retrieve the preconfigured secret key used to encrypt, identified by its fingerprint. Next, we can compute the HMAC of the packet (without the sender HMAC) and check for equality. If the two hash message authentication codes are different, the packet must be dropped, otherwise, the decryption process can begin. First, we have to reconstruct the AES key and IV used to encrypt the message by passing the preconfigured secret key and the message key received in the KDF algorithm presented earlier. Next, we can use the AES 128 algorithm in decrypt mode with the generated key and IV to finally decrypt the whole message. The final step is to remove the padding bytes, which can be easily done with the message size store in the first two bytes.

### 4.2. Tests and Discussion

In order to validate the proposed protocol, we performed three tests. The first one is about bandwidth and attempts to maximize the number of messages per second exchanged to demonstrate the speed of our protocol. The second test answers a question about the UDP protocol. Indeed, UDP does not provide safety mechanisms about lost, corrupted, or disordered network packets. Consequently, we decided to show the number of packets we did not receive because of this insufficiency. Finally, we want to demonstrate the fact that even with the same message and secret key, our encrypted packet is always totally different in order to prevent semantic attack since our initialization vector is not randomly generated. To accomplish this, we will compute a similarity measure between encrypted packets containing the same message with the same encryption key. The hardware used in our tests included a laptop (MSI GT62VR) and Raspberry Pi 3 as well as Raspberry Pi Zero W and a Gigabit wireless router (LinkSys WRT1900AC). The laptop was connected to a Gigabit wireless router (LinkSys WRT1900AC) through its Gigabit wired and wireless (AC Wi-Fi) card and otherwise over a simple Wi-Fi connection. With respect to the implementation of our solution, we used C++ with the libraries Boost ASIO (for the network) and Crypto++ (for the encryption algorithms).

**Figure 7:**
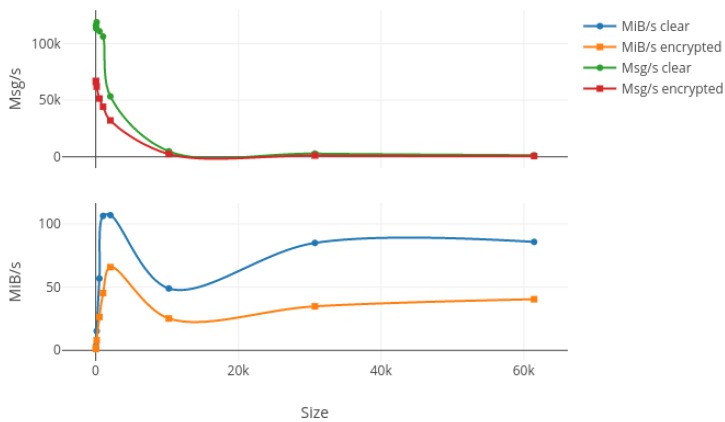
Bandwidth results in terms of Msg/s and MiB/s for 10,000 packets sent on the laptop with increasing message size in both encrypted and clear modes. Msg: messages per second; MiB: mebibytes per second.

**Figure 8:**
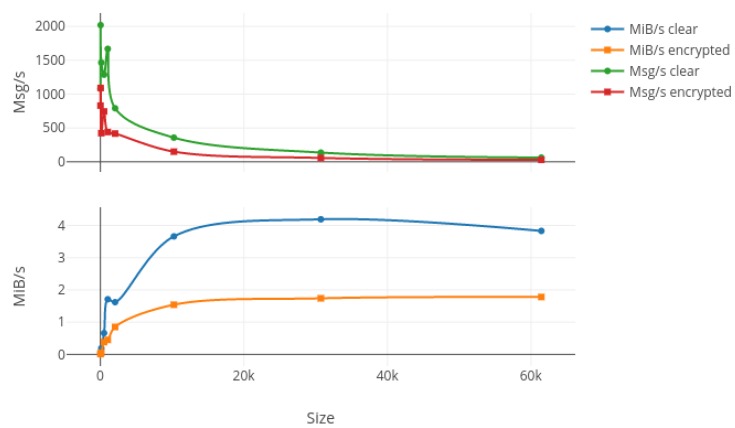
Bandwidth results in terms of Msg/s and MiB/s for 10,000 packets sent on the Raspberry Pi 3 hardware with increasing message size in both encrypted and clear modes.

**Figure 9:**
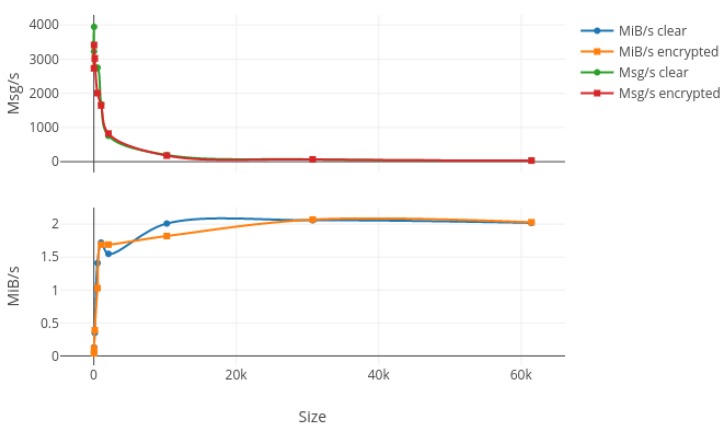
Bandwidth results in terms of Msg/s and MiB/s for 10,000 packets sent on the Raspberry Pi Zero W hardware with increasing message size in both encrypted and clear modes.

In order to test the bandwidth capabilities of our protocol, we first generated nine random messages with different sizes ranging from 16 bytes to 60 kibibytes (kiB). Then, we bound a listening process on the wired network card on the laptop in order to monitor the packets transiting through the network while we used wireless connections to send 20,000 packets for each different size, with 10,000 encrypted and another 10,000 unencrypted. Results from this test are summed up in Figure 7, Figure 8 and Figure 9 where the upper plot represents the messages per second (Msg/s) and the lower one shows the data rate in mebibytes per second (MiB/s) transmitted by our protocol, both depending on the message size. Figure 7, Figure 8 and Figure 9 respectively show the laptop, Raspberry Pi 3 and Raspberry Pi Zero W results. The first characteristic we can note is the important drop in both data rates and message per second occurring for packets larger than 2 kiB on every platform and for both encrypted and non-encrypted messages. Consequently, our solution seems to present a limit concerning high-frequency large messages (greater than 1000 per second). We investigate the reason for such a decrease in performance and it seems to be due to fragmented Ethernet packets that appear when the length of the transported data is greater than the maximum data size of an Ethernet packet, which is 1522 bytes. The second observation is pretty obvious as the message rate and data rate decreased when using encryption. This is due to the computation of different cryptographic algorithms like AES or SHA, but we can say that for a packet under the 2-kiB limit the encryption process does not impact our protocol too greatly. Finally, we can say that our protocol does not overload the Raspberry Pi Zero W platform, which was the smallest, since both encrypted and clear tests give nearly the same results in terms of data rates, which can be explained by a fully-used network adapter.

UDP is a protocol without any reliability mechanisms. This means that packets can be lost or corrupt and the protocol does not support any means to retrieve this packet, unlike Transmission Control Protocol (TCP). As our work is based on UDP and does not provide such mechanisms either, we wanted to show and quantify the risk of data loss. To do so, we create 10,000 messages of 1 kiB composed of a two-byte-long sequence number and some random padding. While the wired interface on the laptop monitors the packet arrival (order, corruption using the embedded checksum, or loss) the wireless connections send 10,000 packets in order. The results of this test are summed up in Table 5 where each line represents a different platform (laptop, Raspberry Pi 3, and Raspberry Pi Zero W) and the three columns are respectively the number of lost packets, corrupted ones, and finally the number of sequencing problems. As we can see, on the same network we did not lose or receive corrupted packets but we had some problems in sequence (two for the laptop and Raspberry Pi 3, and three for Raspberry Pi Zero W). That means that of over 10,000 messages only two or three arrive before the previous ones, a result that tends to prove the relative reliability of our protocol even if we use UDP as our transport protocol.

The last test we performed was a similarity test. Indeed, while we ensure the confidentiality of the data in encrypted mode, we did not randomly generate our initialization vector (IV) for AES encryption. Instead, we use an algorithm (KDF, as described in Algorithm 1) to be able to compute the IV based on information found in the encrypted packet and the secret key. Yet, the randomization of this IV guarantees the semantic security of AES. Consequently, we wanted to know if our algorithm using random padding bytes is random enough to ensure a non-similarity between encrypted packets with the same secret key and containing the same data. To validate this point, we computed the Euclidian distance between 1000 secured packets containing the same 1-kiB message of random data. The results of this test are summed up in Table 6. In this table, the first line represents the maximum distance we can obtain by having one packet with all bytes set to 0 and for the other 255 this maximum distance will be used as a reference to compute the percentage of difference between packets. Next comes the mean distance computed on all the packets, with a value of 3458.79, together with a maximum of 3775.54 and a minimum of 3175.23. We can say that our algorithm guarantees a relative non-similarity between those packets. Finally, we report a mean difference percentage of 42.39% in the last line of the results table. This means that for the exact same packet encrypted with our algorithm we generate encrypted packets that are different by 42.39% on average. Consequently, we can say that our encryption process, even if it does not use a fully random one, computes sufficiently different IVs for the same packet in order to protect it from semantic and similarity attacks.

In conclusion, we can say we performed three main tests. The first one was to compute the speed capabilities of our protocol. In this case the results demonstrate that for data under 2 kiB we assure a very high speed, with nearly 1000 messages per second on the lightest platform we executed the test on. Another conclusion that we came to thanks to this test was the relatively low-impact of the encryption even on the light platform. The second test was performed to ensure the reliability of the UDP multicast on a single network. Indeed, this communication protocol does not provide reliability insurance mechanisms and we wanted to put a number to the risks inherent in its usage. With zero packets lost or corrupted and a maximum of three order problems over 10,000 messages sent we can safely say that even if we use UDP as our base communication protocol, our method seems to be reliable enough for data streaming inside a smart home. Finally, the last test we executed was to compute the similarity between secured packets containing the same message and encrypted with the exact same secret key. Indeed as our initialization vector is computed instead of randomly generated we had to prove that the semantic security of AES is guaranteed. Our results show that our algorithm to derive the AES key and IV from the message itself and the secret pre-shard key is random enough to provide a 42.39% difference on average between 1000 encrypted packets containing the same data.

## 5. Conclusions and Future Works

In this paper, we introduced a new distributed method for communication between smart entities distributed in an environment. Unlike MQTT or RabbitMQ, well-known protocols, we do not need to have a centralized broker instance and unlike ZeroMQ (ZMQ), a well-known framework for implementing messaging protocols, we do not rely on POSIX sockets, which are hard to embed on tiny devices without a Linux or Windows operating system. Our protocol, like FTP, relies on two channels. The first one is for the configuration of each entity in the network and is based on COAP, a well-known protocol in the field of the Internet of things for its reliability. The other is a data channel, based on multicast messages with a protocol fully defined in this paper. This latter channel allows for the sending of discovery requests to the network as well as messages (encrypted or not). For the encrypted method, we adapt the Telegram protocol, a well-known secure instant messaging protocol, in order to work on tiny devices and provide easy authentication with an HMAC.

In this paper, we performed three different tests to ensure our capabilities in terms of speed, reliability, and security. In the light of the results, we can say that for data under 2 kiB we ensure a very high speed, with nearly 1000 messages per second on the lightest platform we executed the test on. Moreover, with no packet lost or corrupted and a maximum of three order problems over 10,000 messages sent we can safely say that our method seems reliable enough for data streaming. Finally, our results show that our algorithm to derive the AES key and IV from the message itself and the secret pre-shard key is random enough to generate a 42.39% difference on average between 1000 encrypted packets containing the same data, ensuring security against semantic attacks.

Ultimately, we can say that our protocol with its two channels allows for making a difference between configuration values, which need high reliability but will not be changing every millisecond, and data values or streaming which need higher data rates but less reliability. Moreover, our protocol supports a native encryption mode which provides security for sensitive information we can easily find in a smart home. In addition, we designed our innovation to be both brokerless, which is the main difference between this and many existing messaging protocols, and easily portable, as it only relies on UDP, a communication protocol found in every network application. Lastly, in future work, we would like to perform more tests, including tests on the embedded environment, like a real-time Operating System (OS) on a micro-controller and tests with many more entities in the network.

## Figures and Tables

**Table 1:** Arduino USB and ESP 32 comparison. BLE: Bluetooth Low Energy.

	Arduino USB	ESP 32 Thing
Released year	2005	2016
Processor frequency	16 MHz	2×240 Mhz
Memory	1 kB	512 kB
Connectivity	None	Bluetooth + BLE and WiFi
Relative size	1	0.5
Price (USD)	35	7

**Table 2:** The Raspberry Pi platform over time.

	Pi 1	Pi Zero W	Pi 3
Released year	2012	2017	2016
Processor frequency	700 MHz	1 GHz	4×1.2 GHz
Memory	512 MB	512 MB	1 GB
Connectivity	None	BLE/WiFi	BLE/WiFi
Relative size	1	0.5	1
Price (USD)	35	9	35

**Table 3:** A configuration example based on the Constrained Application Protocol (CoAP). URI : Unified Resource Identifier, JSON : JavaScript Object Notation.

	URI	Methods Allowed	Data Type
Update rate	/rate	GET/POST	Integer
Version	/version	GET	String
Hardware	/hardware	GET/POST	JSON
Update	/update	POST	Binary
Encryption key	/keys	POST	Binary

**Table 4:** An example of the discovery decision process.

Key	Requested	Exposed	Conclusion
version	“1.0.1”	“1.0.1”	Answer
name	“temp2”	“temp32”	No answer
units	“C”	[“C”, “F”]	Answer
units	“K”	[“C”, “F”]	No answer
units	[“C”, “F”]	“C”	Answer
units	[“C”, “F”]	“K”	No answer
units	[“C”, “F”]	[“C”, “K”]	Answer
units	[“C”, “F”]	[“K”, “R”]	No answer

**Table 5:** Number of lost, corrupted or misplaced packets for over 10,000 sent when using the 1-kiB generated packet.

Sender	Packets Lost	Packets Corrupted	Sequence Problem
Laptop	0	0	2
Pi 3	0	0	2
Pi Zero W	0	0	3

**Table 6:** Distance between the same 1000 1-kiB packets on the same topic encrypted with the same encryption key.

Variable	Value
Maximum theoretical distance	8160
Mean distance	3458.79
Maximum distance	3775.54
Minimum distance	3175.23
Mean difference percentage	42.39%

## Abbreviations

The following abbreviations are used in this manuscript:

AES	Advanced Encryption Standard
AI	artificial intelligence
Amb. I	ambient intelligence
AMQP	Advanced Message Queuing Protocol
BLE	Bluetooth Low Energy
CoAP	Constrained Application Protocol
CRC	cyclic redundancy check
EEPROM	electrically erasable programmable read-only memory
GHz	gigahertz
HMAC	keyed-hash message authentication code
HTTP	Hypertext Transfer Protocol
IEEE	Institute of Electrical and Electronics Engineers
IoT	Internet of things
IV	initialization vector
IP	Internet Protocol
KDF	key derivation function
LIARA	*Laboratoire d’Intelligence Ambiante pour la Reconnaissance d’Activités*
LSB	least significant bit
LWT	last will testament
kB	kilobyte
kiB	kibibyte
kiB/s	kibibyte/second
MHz	megahertz
MiB	mebibyte
MiB/s	mebibyte/second
MQTT	Message Queue Telemetry Transport
MSB	most significant bit
Msg/s	messages/seconds
OSGI	Open Service Gateway initiative
POSIX	Portable Operating System Interface
RAM	Random Access Memory
RTOS	real-time operating system
SDSH	software-defined smart home
SHA	Secure Hash Algorithms
SoC	system-on-chip
SPoF	single point of failure
UDP	User Datagram Protocol
XMPP	eXtensible Messaging and Presence Protocol
ZMQ	ZeroMQ
